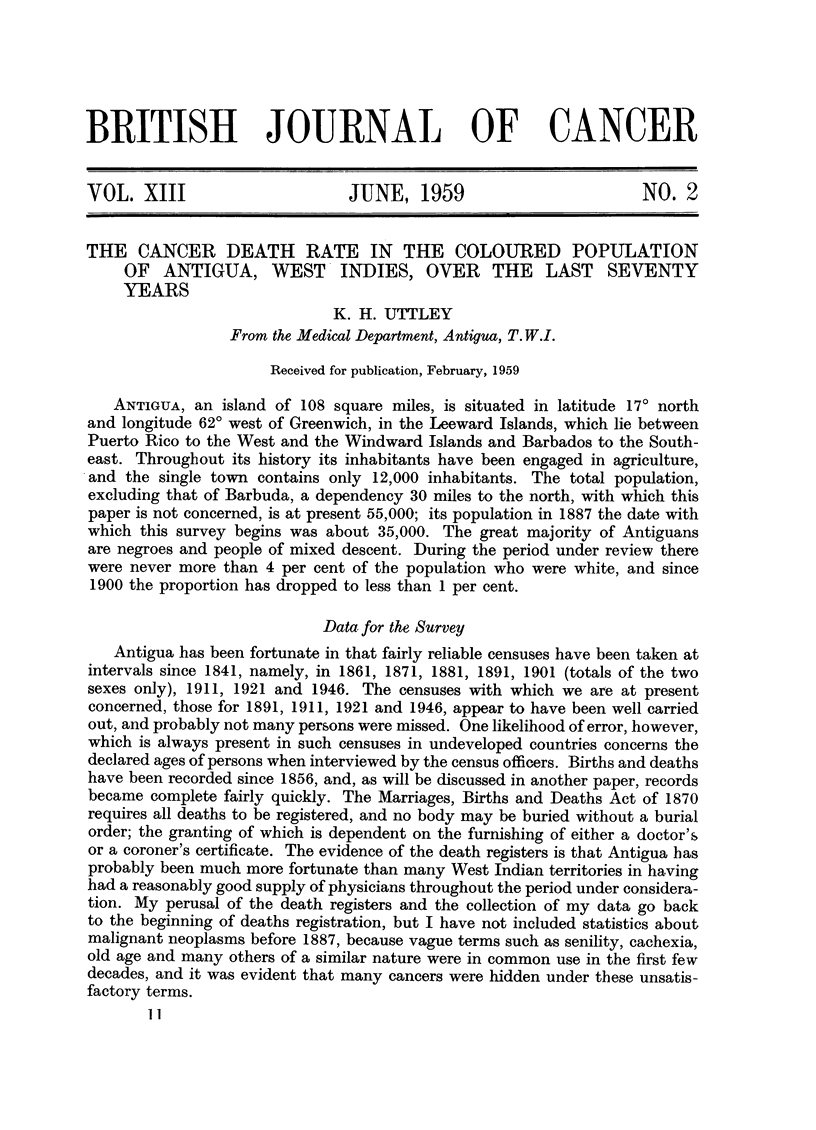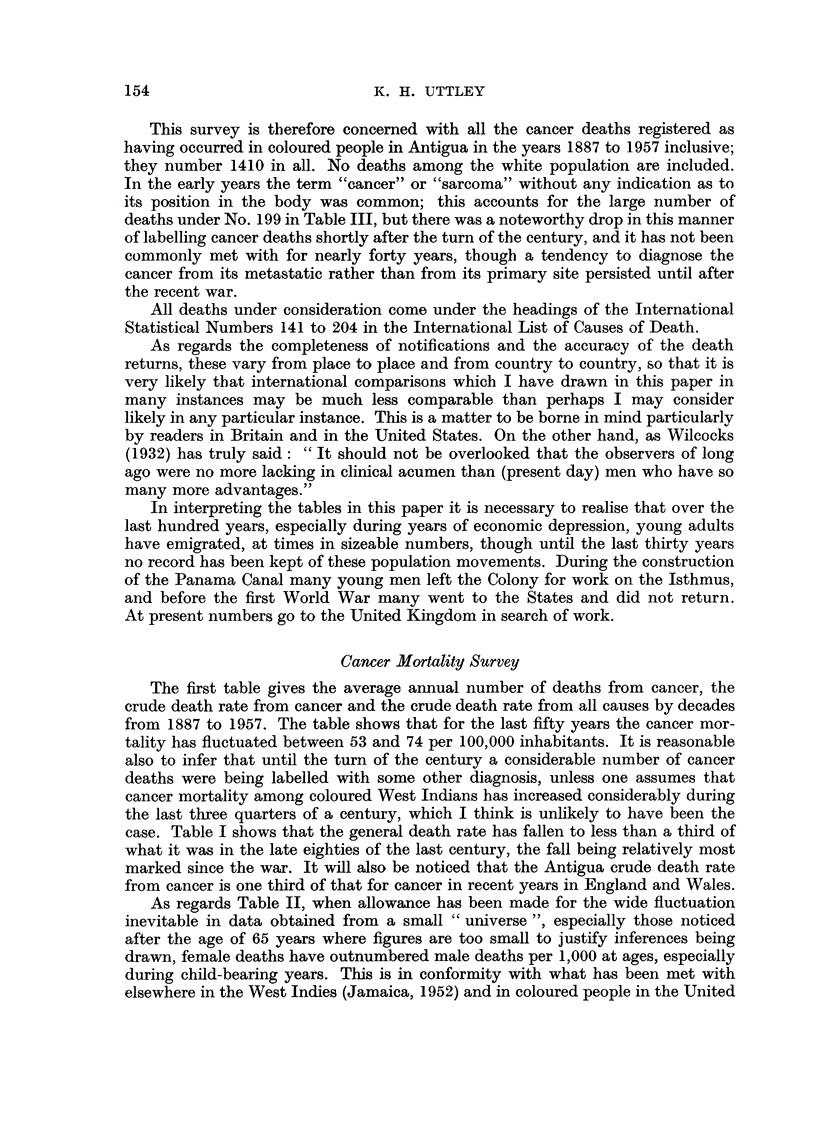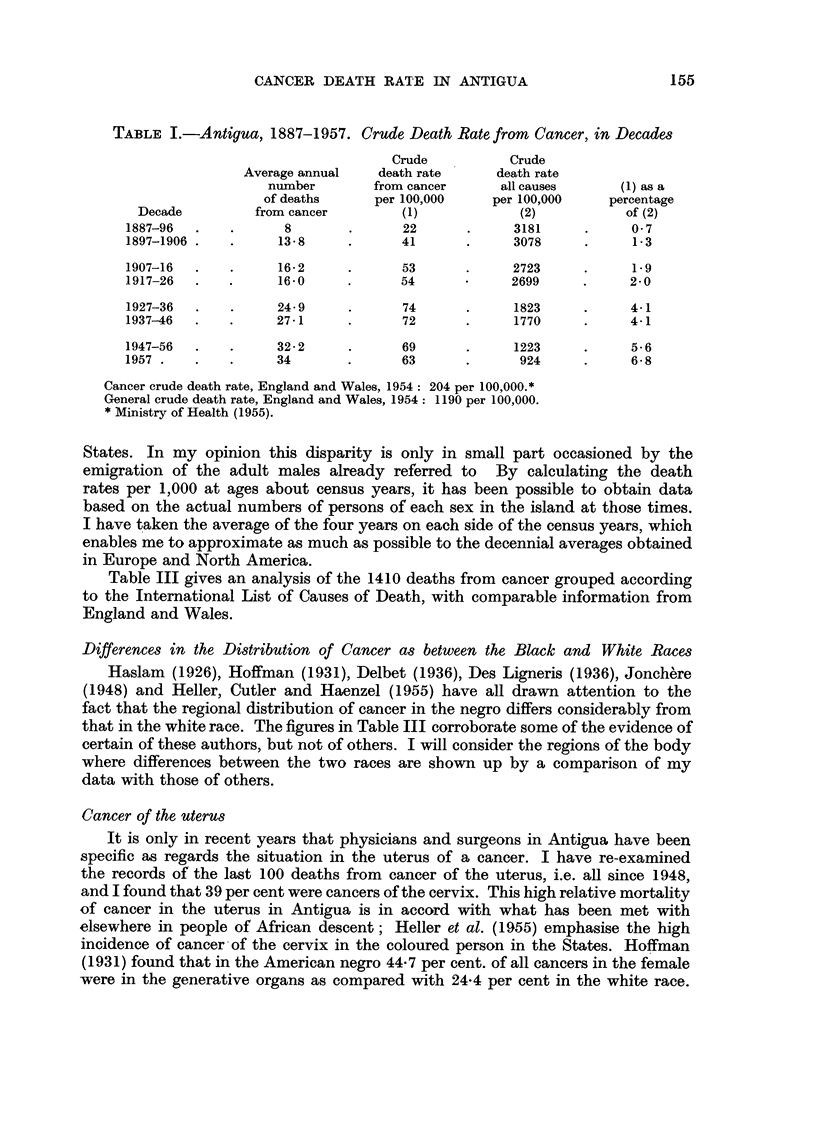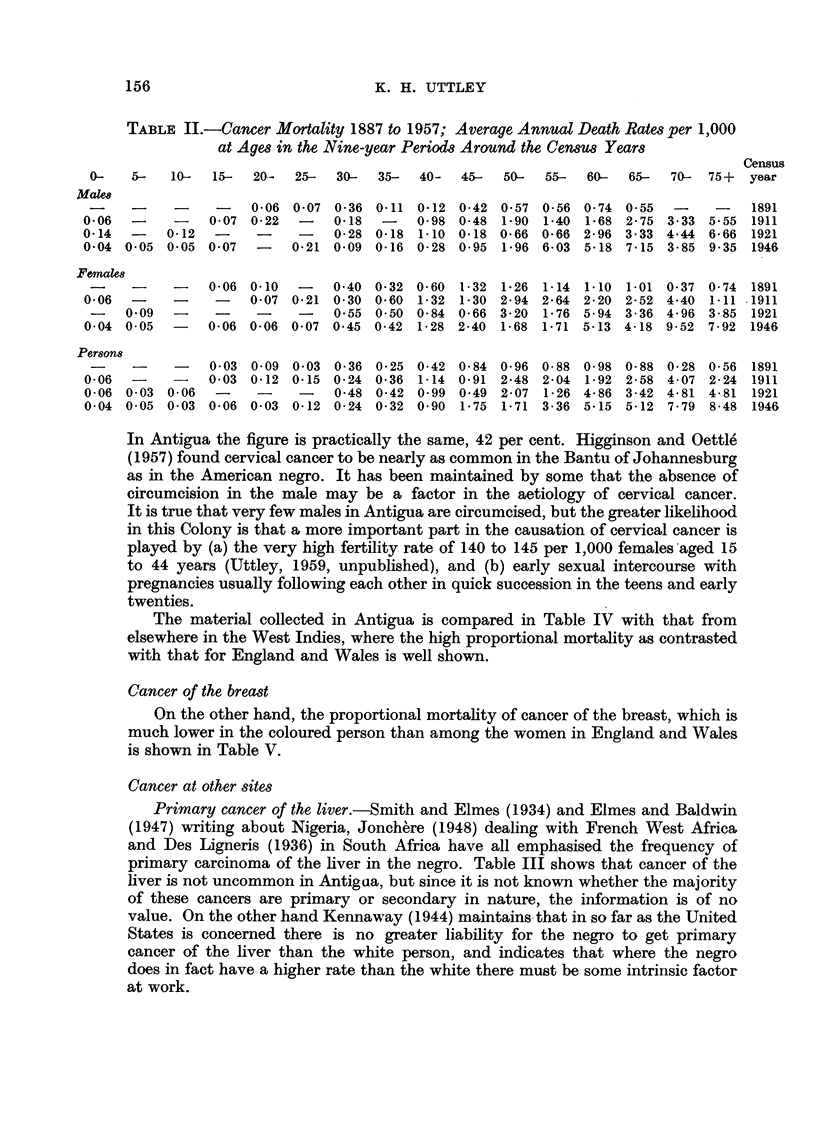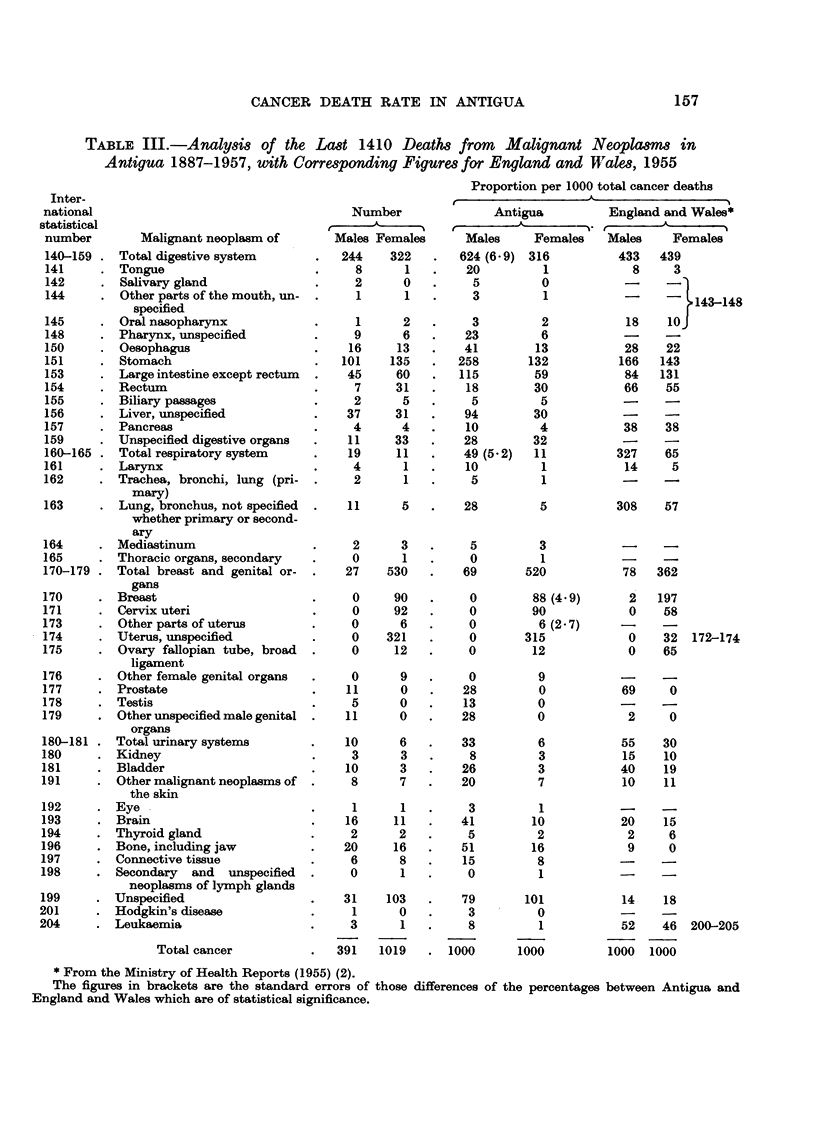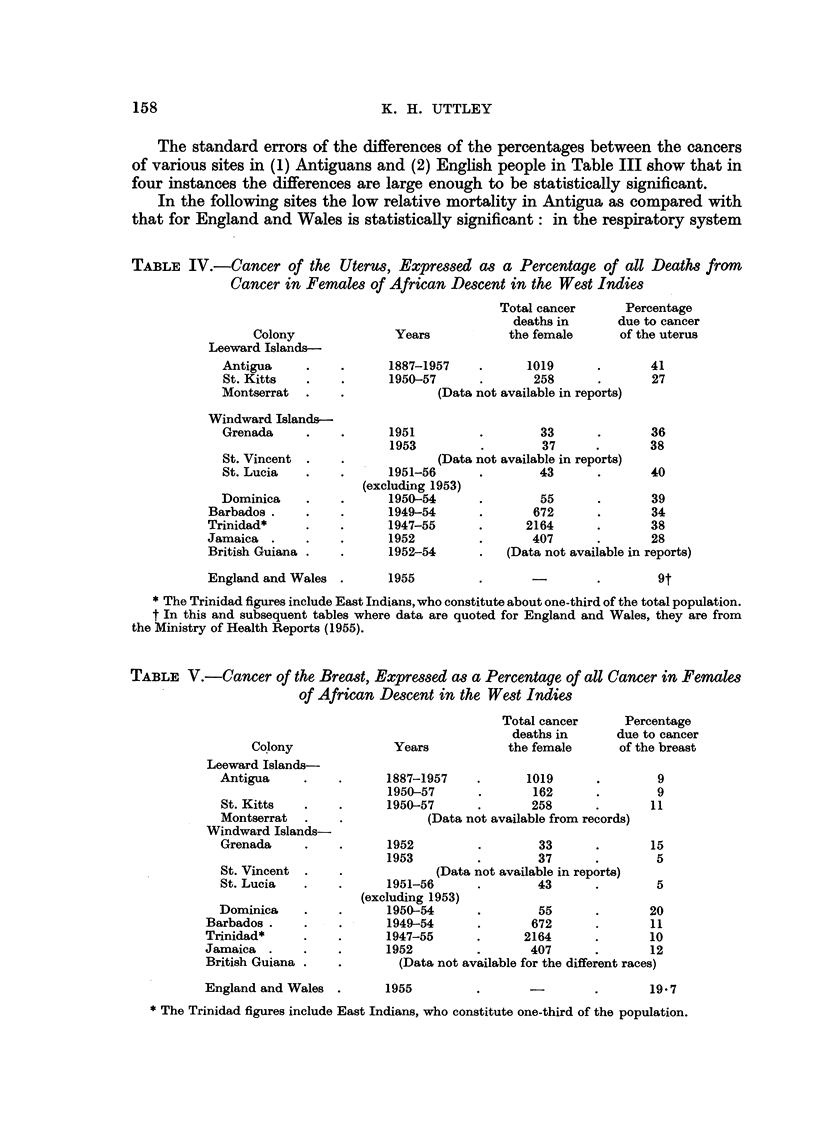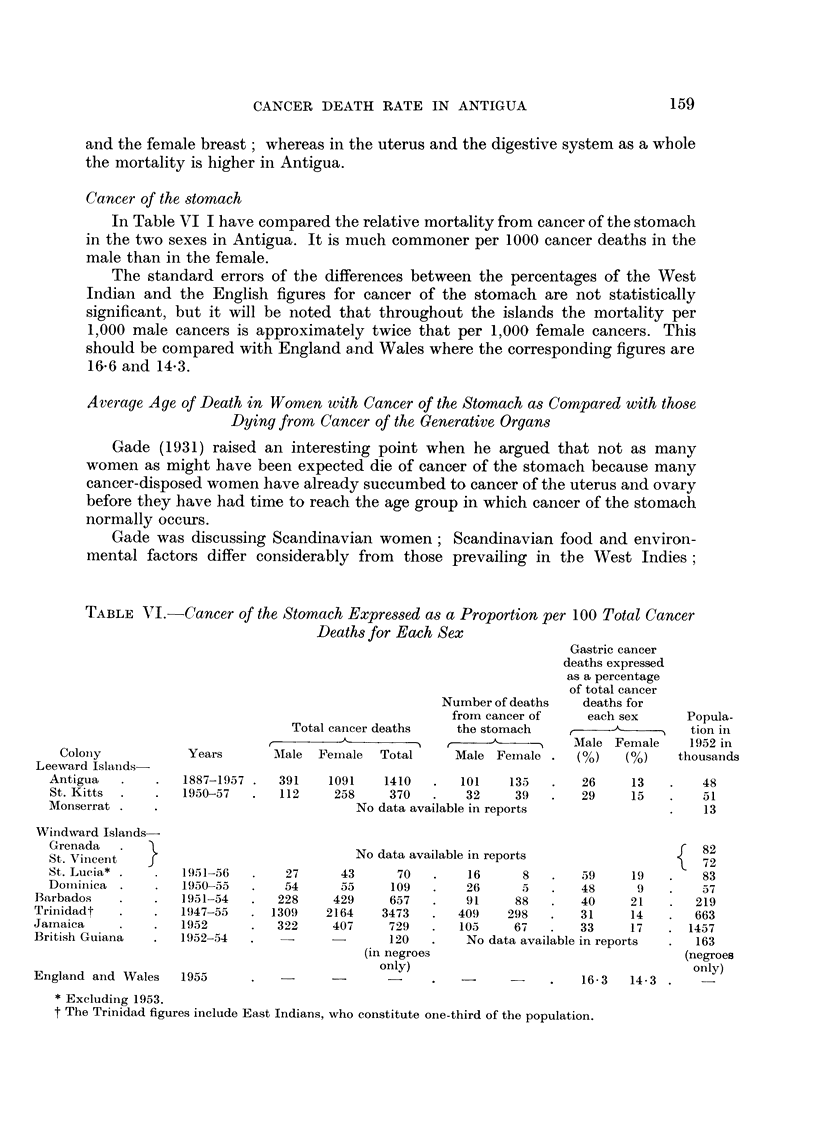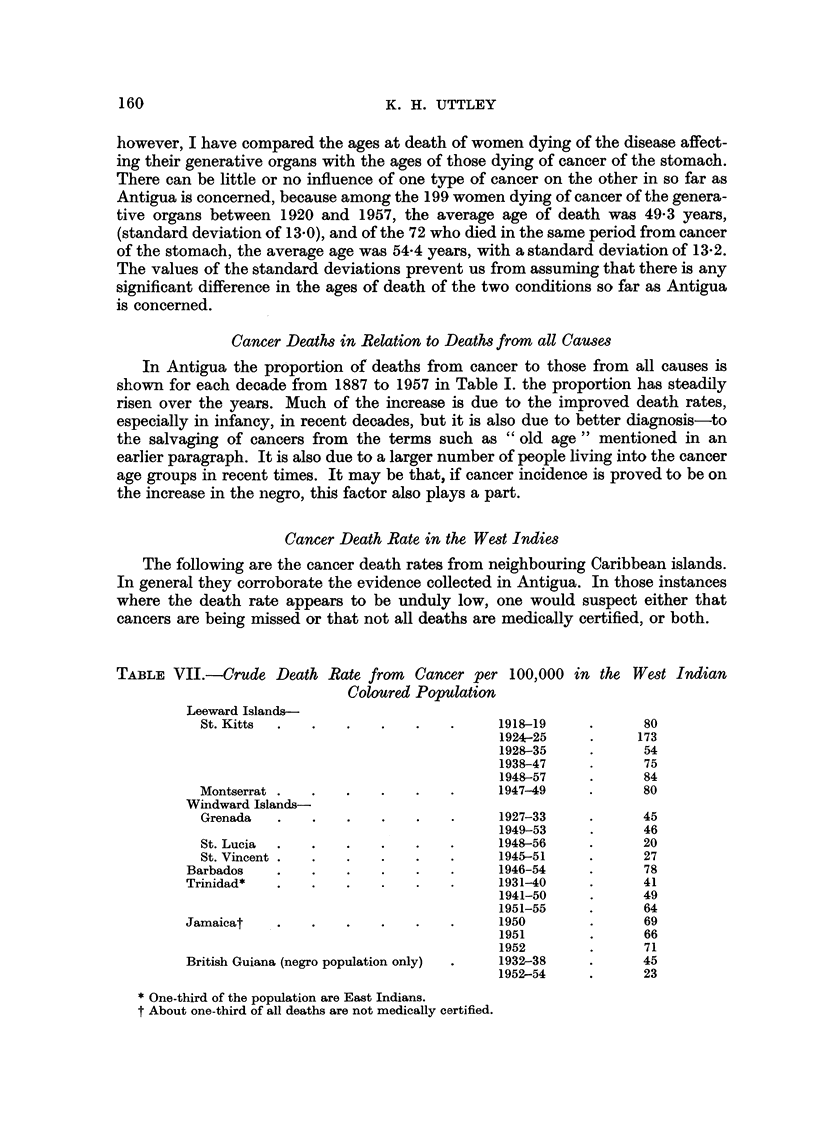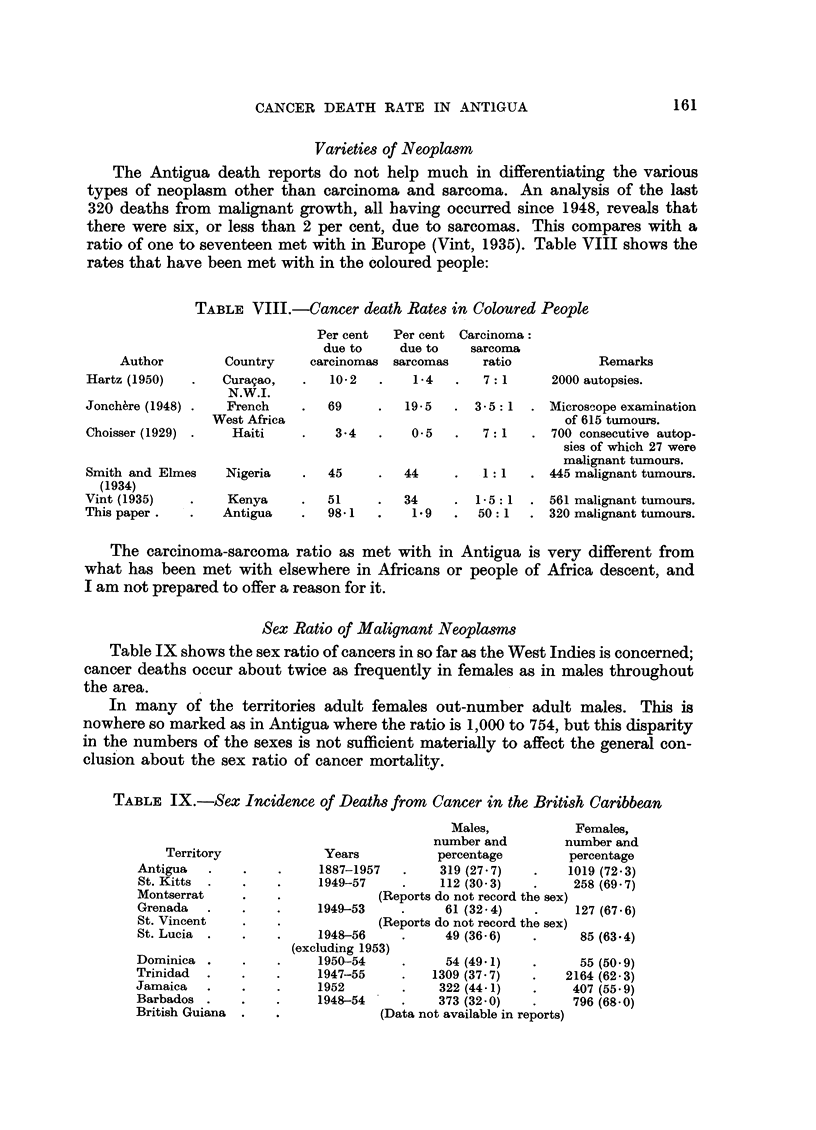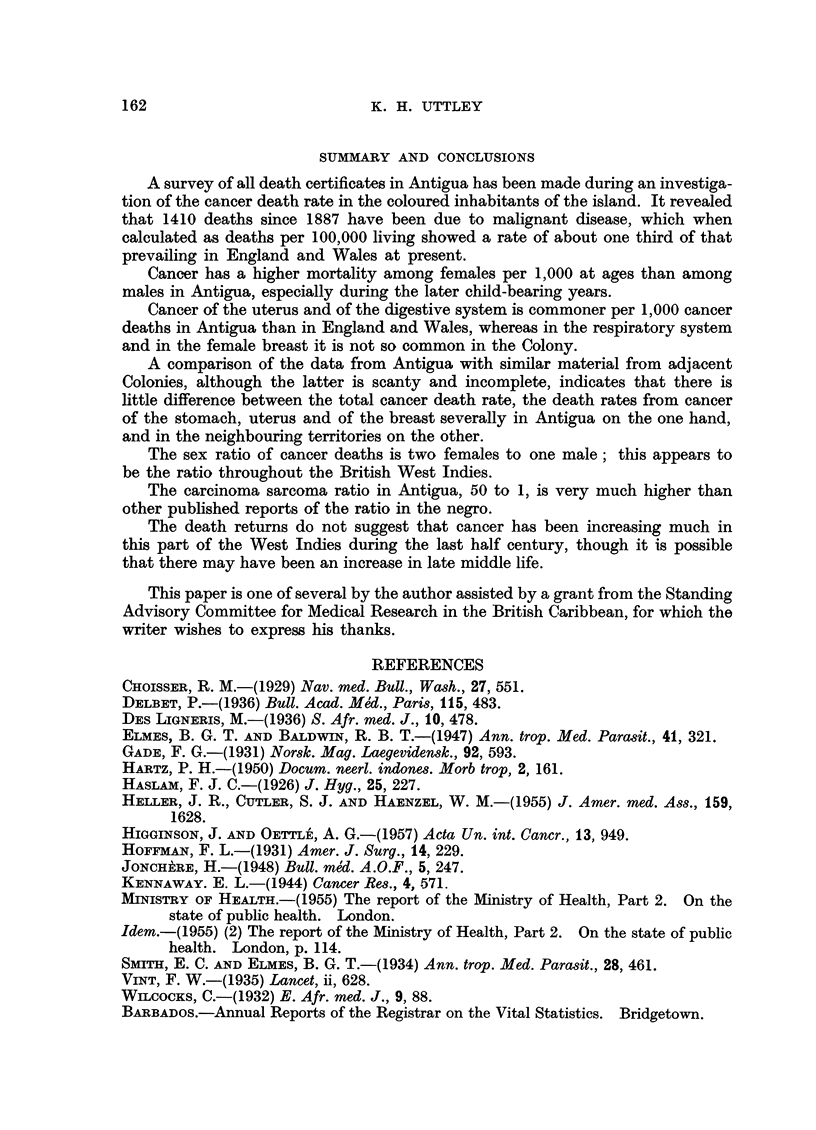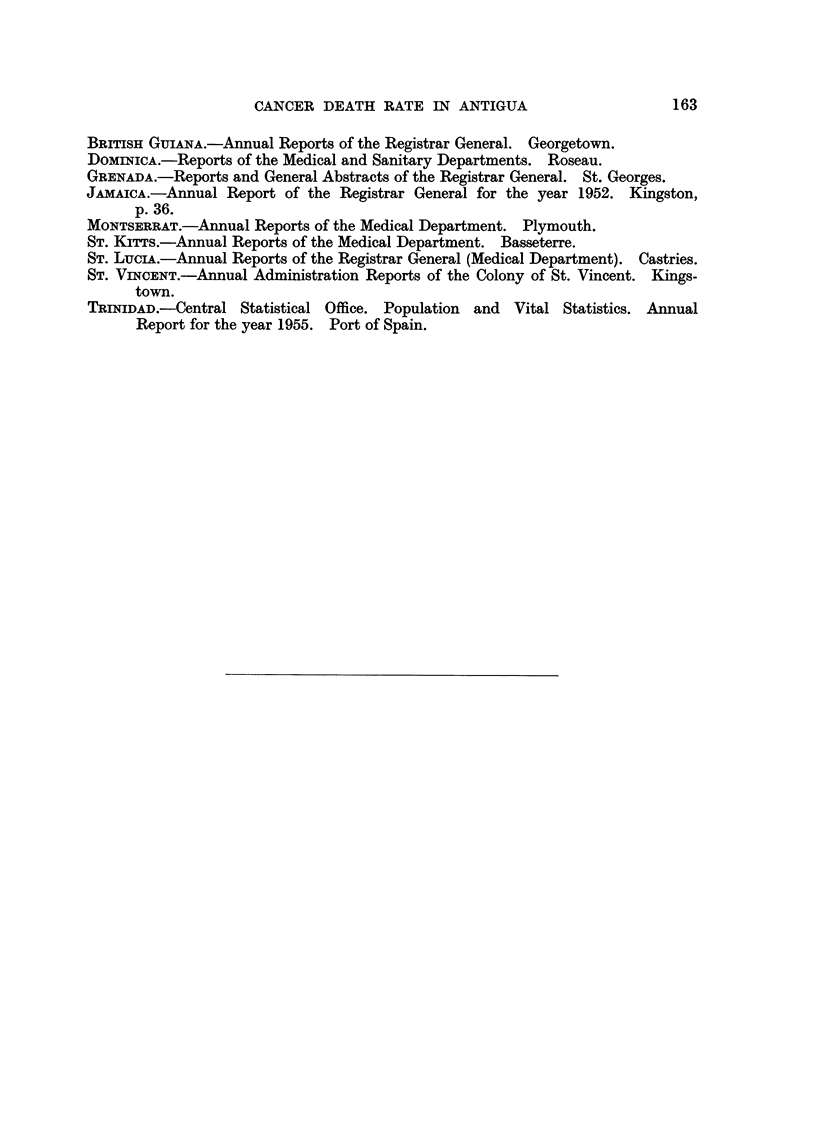# The Cancer Death Rate in the Coloured Population of Antigua, West Indies, Over the Last Seventy Years

**DOI:** 10.1038/bjc.1959.21

**Published:** 1959-06

**Authors:** K. H. Uttley


BRITISH JOURNAL OF CANCER

VOL. XIII JUNE, 1959 NO. 2

### THE CANCER DEATH RATE IN THE COLOURED POPULATION OF ANTIGUA, WEST INDIES, OVER THE LAST SEVENTY YEARS

K. H. UTTLEY

*From the Medical Department, Antigua, T.W.I.*

Received for publication, February, 1959

ANTIGUA, an island of 108 square miles, is situated in latitude 17° north and longitude 62° west of Greenwich, in the Leeward Islands, which lie between Puerto Rico to the West and the Windward Islands and Barbados to the South-east. Throughout its history its inhabitants have been engaged in agriculture, and the single town contains only 12,000 inhabitants. The total population, excluding that of Barbuda, a dependency 30 miles to the north, with which this paper is not concerned, is at present 55,000; its population in 1887 the date with which this survey begins was about 35,000. The great majority of Antiguans are negroes and people of mixed descent. During the period under review there were never more than 4 per cent of the population who were white, and since 1900 the proportion has dropped to less than 1 per cent.

### *Data for the Survey*

Antigua has been fortunate in that fairly reliable censuses have been taken at intervals since 1841, namely, in 1861, 1871, 1881, 1891, 1901 (totals of the two sexes only), 1911, 1921 and 1946. The censuses with which we are at present concerned, those for 1891, 1911, 1921 and 1946, appear to have been well carried out, and probably not many persons were missed. One likelihood of error, however, which is always present in such censuses in undeveloped countries concerns the declared ages of persons when interviewed by the census officers. Births and deaths have been recorded since 1856, and, as will be discussed in another paper, records became complete fairly quickly. The Marriages, Births and Deaths Act of 1870 requires all deaths to be registered, and no body may be buried without a burial order; the granting of which is dependent on the furnishing of either a doctor's or a coroner's certificate. The evidence of the death registers is that Antigua has probably been much more fortunate than many West Indian territories in having had a reasonably good supply of physicians throughout the period under consideration. My perusal of the death registers and the collection of my data go back to the beginning of deaths registration, but I have not included statistics about malignant neoplasms before 1887, because vague terms such as senility, cachexia, old age and many others of a similar nature were in common use in the first few decades, and it was evident that many cancers were hidden under these unsatisfactory terms.

This survey is therefore concerned with all the cancer deaths registered as having occurred in coloured people in Antigua in the years 1887 to 1957 inclusive; they number 1410 in all. No deaths among the white population are included. In the early years the term "cancer" or "sarcoma" without any indication as to its position in the body was common; this accounts for the large number of deaths under No. 199 in Table III, but there was a noteworthy drop in this manner of labelling cancer deaths shortly after the turn of the century, and it has not been commonly met with for nearly forty years, though a tendency to diagnose the cancer from its metastatic rather than from its primary site persisted until after the recent war.

All deaths under consideration come under the headings of the International Statistical Numbers 141 to 204 in the International List of Causes of Death.

As regards the completeness of notifications and the accuracy of the death returns, these vary from place to place and from country to country, so that it is very likely that international comparisons which I have drawn in this paper in many instances may be much less comparable than perhaps I may consider likely in any particular instance. This is a matter to be borne in mind particularly by readers in Britain and in the United States. On the other hand, as Wilcocks (1932) has truly said : " It should not be overlooked that the observers of long ago were no more lacking in clinical acumen than (present day) men who have so many more advantages."

In interpreting the tables in this paper it is necessary to realise that over the last hundred years, especially during years of economic depression, young adults have emigrated, at times in sizeable numbers, though until the last thirty years no record has been kept of these population movements. During the construction of the Panama Canal many young men left the Colony for work on the Isthmus, and before the first World War many went to the States and did not return. At present numbers go to the United Kingdom in search of work.

### *Cancer Mortality Survey*

The first table gives the average annual number of deaths from cancer, the crude death rate from cancer and the crude death rate from all causes by decades from 1887 to 1957. The table shows that for the last fifty years the cancer mortality has fluctuated between 53 and 74 per 100,000 inhabitants. It is reasonable also to infer that until the turn of the century a considerable number of cancer deaths were being labelled with some other diagnosis, unless one assumes that cancer mortality among coloured West Indians has increased considerably during the last three quarters of a century, which I think is unlikely to have been the case. Table I shows that the general death rate has fallen to less than a third of what it was in the late eighties of the last century, the fall being relatively most marked since the war. It will also be noticed that the Antigua crude death rate from cancer is one third of that for cancer in recent years in England and Wales.

As regards Table II, when allowance has been made for the wide fluctuation inevitable in data obtained from a small " universe ", especially those noticed after the age of 65 years where figures are too small to justify inferences being drawn, female deaths have outnumbered male deaths per 1,000 at ages, especially during child-bearing years. This is in conformity with what has been met with elsewhere in the West Indies (Jamaica, 1952) and in coloured people in the United

TABLE I.—*Antigua*, 1887–1957. *Crude Death Rate from Cancer, in Decades*

| Decade | Average annual number of deaths from cancer | Crude death rate from cancer per 100,000 (1) | Crude death rate all causes per 100,000 (2) | (1) as a percentage of (2) |
|---|---|---|---|---|
| 1887–96 | 8 | 22 | 3181 | 0·7 |
| 1897–1906 | 13·8 | 41 | 3078 | 1·3 |
| 1907–16 | 16·2 | 53 | 2723 | 1·9 |
| 1917–26 | 16·0 | 54 | 2699 | 2·0 |
| 1927–36 | 24·9 | 74 | 1823 | 4·1 |
| 1937–46 | 27·1 | 72 | 1770 | 4·1 |
| 1947–56 | 32·2 | 69 | 1223 | 5·6 |
| 1957 | 34 | 63 | 924 | 6·8 |

Cancer crude death rate, England and Wales, 1954 : 204 per 100,000.*
General crude death rate, England and Wales, 1954 : 1190 per 100,000.
* Ministry of Health (1955).

States. In my opinion this disparity is only in small part occasioned by the emigration of the adult males already referred to By calculating the death rates per 1,000 at ages about census years, it has been possible to obtain data based on the actual numbers of persons of each sex in the island at those times. I have taken the average of the four years on each side of the census years, which enables me to approximate as much as possible to the decennial averages obtained in Europe and North America.

Table III gives an analysis of the 1410 deaths from cancer grouped according to the International List of Causes of Death, with comparable information from England and Wales.

*Differences in the Distribution of Cancer as between the Black and White Races*

Haslam (1926), Hoffman (1931), Delbet (1936), Des Ligneris (1936), Jonchère (1948) and Heller, Cutler and Haenzel (1955) have all drawn attention to the fact that the regional distribution of cancer in the negro differs considerably from that in the white race. The figures in Table III corroborate some of the evidence of certain of these authors, but not of others. I will consider the regions of the body where differences between the two races are shown up by a comparison of my data with those of others.

*Cancer of the uterus*

It is only in recent years that physicians and surgeons in Antigua have been specific as regards the situation in the uterus of a cancer. I have re-examined the records of the last 100 deaths from cancer of the uterus, i.e. all since 1948, and I found that 39 per cent were cancers of the cervix. This high relative mortality of cancer in the uterus in Antigua is in accord with what has been met with elsewhere in people of African descent ; Heller *et al.* (1955) emphasise the high incidence of cancer of the cervix in the coloured person in the States. Hoffman (1931) found that in the American negro 44·7 per cent. of all cancers in the female were in the generative organs as compared with 24·4 per cent in the white race.

TABLE II.—*Cancer Mortality* 1887 *to* 1957; *Average Annual Death Rates per* 1,000 *at Ages in the Nine-year Periods Around the Census Years*

| 0– | 5– | 10– | 15– | 20– | 25– | 30– | 35– | 40– | 45– | 50– | 55– | 60– | 65– | 70– | 75+ | Census year |
|---|---|---|---|---|---|---|---|---|---|---|---|---|---|---|---|---|
| *Males* | | | | | | | | | | | | | | | | |
| — | — | — | — | 0·06 | 0·07 | 0·36 | 0·11 | 0·12 | 0·42 | 0·57 | 0·56 | 0·74 | 0·55 | — | — | 1891 |
| 0·06 | — | — | 0·07 | 0·22 | — | 0·18 | — | 0·98 | 0·48 | 1·90 | 1·40 | 1·68 | 2·75 | 3·33 | 5·55 | 1911 |
| 0·14 | — | 0·12 | — | — | — | 0·28 | 0·18 | 1·10 | 0·18 | 0·66 | 0·66 | 2·96 | 3·33 | 4·44 | 6·66 | 1921 |
| 0·04 | 0·05 | 0·05 | 0·07 | — | 0·21 | 0·09 | 0·16 | 0·28 | 0·95 | 1·96 | 6·03 | 5·18 | 7·15 | 3·85 | 9·35 | 1946 |
| *Females* | | | | | | | | | | | | | | | | |
| — | — | — | 0·06 | 0·10 | — | 0·40 | 0·32 | 0·60 | 1·32 | 1·26 | 1·14 | 1·10 | 1·01 | 0·37 | 0·74 | 1891 |
| 0·06 | — | — | — | 0·07 | 0·21 | 0·30 | 0·60 | 1·32 | 1·30 | 2·94 | 2·64 | 2·20 | 2·52 | 4·40 | 1·11 | 1911 |
| — | 0·09 | — | — | — | — | 0·55 | 0·50 | 0·84 | 0·66 | 3·20 | 1·76 | 5·94 | 3·36 | 4·96 | 3·85 | 1921 |
| 0·04 | 0·05 | — | 0·06 | 0·06 | 0·07 | 0·45 | 0·42 | 1·28 | 2·40 | 1·68 | 1·71 | 5·13 | 4·18 | 9·52 | 7·92 | 1946 |
| *Persons* | | | | | | | | | | | | | | | | |
| — | — | — | 0·03 | 0·09 | 0·03 | 0·36 | 0·25 | 0·42 | 0·84 | 0·96 | 0·88 | 0·98 | 0·88 | 0·28 | 0·56 | 1891 |
| 0·06 | — | — | 0·03 | 0·12 | 0·15 | 0·24 | 0·36 | 1·14 | 0·91 | 2·48 | 2·04 | 1·92 | 2·58 | 4·07 | 2·24 | 1911 |
| 0·06 | 0·03 | 0·06 | — | — | — | 0·48 | 0·42 | 0·99 | 0·49 | 2·07 | 1·26 | 4·86 | 3·42 | 4·81 | 4·81 | 1921 |
| 0·04 | 0·05 | 0·03 | 0·06 | 0·03 | 0·12 | 0·24 | 0·32 | 0·90 | 1·75 | 1·71 | 3·36 | 5·15 | 5·12 | 7·79 | 8·48 | 1946 |

In Antigua the figure is practically the same, 42 per cent. Higginson and Oettlé (1957) found cervical cancer to be nearly as common in the Bantu of Johannesburg as in the American negro. It has been maintained by some that the absence of circumcision in the male may be a factor in the aetiology of cervical cancer. It is true that very few males in Antigua are circumcised, but the greater likelihood in this Colony is that a more important part in the causation of cervical cancer is played by (a) the very high fertility rate of 140 to 145 per 1,000 females aged 15 to 44 years (Uttley, 1959, unpublished), and (b) early sexual intercourse with pregnancies usually following each other in quick succession in the teens and early twenties.

The material collected in Antigua is compared in Table IV with that from elsewhere in the West Indies, where the high proportional mortality as contrasted with that for England and Wales is well shown.

*Cancer of the breast*

On the other hand, the proportional mortality of cancer of the breast, which is much lower in the coloured person than among the women in England and Wales is shown in Table V.

*Cancer at other sites*

*Primary cancer of the liver.*—Smith and Elmes (1934) and Elmes and Baldwin (1947) writing about Nigeria, Jonchère (1948) dealing with French West Africa and Des Ligneris (1936) in South Africa have all emphasised the frequency of primary carcinoma of the liver in the negro. Table III shows that cancer of the liver is not uncommon in Antigua, but since it is not known whether the majority of these cancers are primary or secondary in nature, the information is of no value. On the other hand Kennaway (1944) maintains that in so far as the United States is concerned there is no greater liability for the negro to get primary cancer of the liver than the white person, and indicates that where the negro does in fact have a higher rate than the white there must be some intrinsic factor at work.

TABLE III.—*Analysis of the Last* 1410 *Deaths from Malignant Neoplasms in Antigua* 1887–1957, *with Corresponding Figures for England and Wales*, 1955

| International statistical number | Malignant neoplasm of | Number | | Proportion per 1000 total cancer deaths: Antigua | | England and Wales* | | |
|---|---|---|---|---|---|---|---|---|
| | | Males | Females | Males | Females | Males | Females | |
| 140–159 | Total digestive system | 244 | 322 | 624 (6·9) | 316 | 433 | 439 | |
| 141 | Tongue | 8 | 1 | 20 | 1 | 8 | 3 | |
| 142 | Salivary gland | 2 | 0 | 5 | 0 | — | — | 143–148 |
| 144 | Other parts of the mouth, unspecified | 1 | 1 | 3 | 1 | — | — | |
| 145 | Oral nasopharynx | 1 | 2 | 3 | 2 | 18 | 10 | |
| 148 | Pharynx, unspecified | 9 | 6 | 23 | 6 | — | — | |
| 150 | Oesophagus | 16 | 13 | 41 | 13 | 28 | 22 | |
| 151 | Stomach | 101 | 135 | 258 | 132 | 166 | 143 | |
| 153 | Large intestine except rectum | 45 | 60 | 115 | 59 | 84 | 131 | |
| 154 | Rectum | 7 | 31 | 18 | 30 | 66 | 55 | |
| 155 | Biliary passages | 2 | 5 | 5 | 5 | — | — | |
| 156 | Liver, unspecified | 37 | 31 | 94 | 30 | — | — | |
| 157 | Pancreas | 4 | 4 | 10 | 4 | 38 | 38 | |
| 159 | Unspecified digestive organs | 11 | 33 | 28 | 32 | — | — | |
| 160–165 | Total respiratory system | 19 | 11 | 49 (5·2) | 11 | 327 | 65 | |
| 161 | Larynx | 4 | 1 | 10 | 1 | 14 | 5 | |
| 162 | Trachea, bronchi, lung (primary) | 2 | 1 | 5 | 1 | — | — | |
| 163 | Lung, bronchus, not specified whether primary or secondary | 11 | 5 | 28 | 5 | 308 | 57 | |
| 164 | Mediastinum | 2 | 3 | 5 | 3 | — | — | |
| 165 | Thoracic organs, secondary | 0 | 1 | 0 | 1 | — | — | |
| 170–179 | Total breast and genital organs | 27 | 530 | 69 | 520 | 78 | 362 | |
| 170 | Breast | 0 | 90 | 0 | 88 (4·9) | 2 | 197 | |
| 171 | Cervix uteri | 0 | 92 | 0 | 90 | 0 | 58 | |
| 173 | Other parts of uterus | 0 | 6 | 0 | 6 (2·7) | — | — | |
| 174 | Uterus, unspecified | 0 | 321 | 0 | 315 | 0 | 32 | 172–174 |
| 175 | Ovary fallopian tube, broad ligament | 0 | 12 | 0 | 12 | 0 | 65 | |
| 176 | Other female genital organs | 0 | 9 | 0 | 9 | — | — | |
| 177 | Prostate | 11 | 0 | 28 | 0 | 69 | 0 | |
| 178 | Testis | 5 | 0 | 13 | 0 | — | — | |
| 179 | Other unspecified male genital organs | 11 | 0 | 28 | 0 | 2 | 0 | |
| 180–181 | Total urinary systems | 10 | 6 | 33 | 6 | 55 | 30 | |
| 180 | Kidney | 3 | 3 | 8 | 3 | 15 | 10 | |
| 181 | Bladder | 10 | 3 | 26 | 3 | 40 | 19 | |
| 191 | Other malignant neoplasms of the skin | 8 | 7 | 20 | 7 | 10 | 11 | |
| 192 | Eye | 1 | 1 | 3 | 1 | — | — | |
| 193 | Brain | 16 | 11 | 41 | 10 | 20 | 15 | |
| 194 | Thyroid gland | 2 | 2 | 5 | 2 | 2 | 6 | |
| 196 | Bone, including jaw | 20 | 16 | 51 | 16 | 9 | 0 | |
| 197 | Connective tissue | 6 | 8 | 15 | 8 | — | — | |
| 198 | Secondary and unspecified neoplasms of lymph glands | 0 | 1 | 0 | 1 | — | — | |
| 199 | Unspecified | 31 | 103 | 79 | 101 | 14 | 18 | |
| 201 | Hodgkin's disease | 1 | 0 | 3 | 0 | — | — | |
| 204 | Leukaemia | 3 | 1 | 8 | 1 | 52 | 46 | 200–205 |
| | Total cancer | 391 | 1019 | 1000 | 1000 | 1000 | 1000 | |

* From the Ministry of Health Reports (1955) (2).

The figures in brackets are the standard errors of those differences of the percentages between Antigua and England and Wales which are of statistical significance.

The standard errors of the differences of the percentages between the cancers of various sites in (1) Antiguans and (2) English people in Table III show that in four instances the differences are large enough to be statistically significant.

In the following sites the low relative mortality in Antigua as compared with that for England and Wales is statistically significant : in the respiratory system

TABLE IV.—*Cancer of the Uterus, Expressed as a Percentage of all Deaths from Cancer in Females of African Descent in the West Indies*

| Colony | Years | Total cancer deaths in the female | Percentage due to cancer of the uterus |
|---|---|---|---|
| Leeward Islands— | | | |
| Antigua | 1887–1957 | 1019 | 41 |
| St. Kitts | 1950–57 | 258 | 27 |
| Montserrat | (Data not available in reports) | | |
| Windward Islands— | | | |
| Grenada | 1951 | 33 | 36 |
| | 1953 | 37 | 38 |
| St. Vincent | (Data not available in reports) | | |
| St. Lucia | 1951–56 (excluding 1953) | 43 | 40 |
| Dominica | 1950–54 | 55 | 39 |
| Barbados | 1949–54 | 672 | 34 |
| Trinidad* | 1947–55 | 2164 | 38 |
| Jamaica | 1952 | 407 | 28 |
| British Guiana | 1952–54 | (Data not available in reports) | |
| England and Wales | 1955 | — | 9† |

* The Trinidad figures include East Indians, who constitute about one-third of the total population.
† In this and subsequent tables where data are quoted for England and Wales, they are from the Ministry of Health Reports (1955).

TABLE V.—*Cancer of the Breast, Expressed as a Percentage of all Cancer in Females of African Descent in the West Indies*

| Colony | Years | Total cancer deaths in the female | Percentage due to cancer of the breast |
|---|---|---|---|
| Leeward Islands— | | | |
| Antigua | 1887–1957 | 1019 | 9 |
| | 1950–57 | 162 | 9 |
| St. Kitts | 1950–57 | 258 | 11 |
| Montserrat | (Data not available from records) | | |
| Windward Islands— | | | |
| Grenada | 1952 | 33 | 15 |
| | 1953 | 37 | 5 |
| St. Vincent | (Data not available in reports) | | |
| St. Lucia | 1951–56 (excluding 1953) | 43 | 5 |
| Dominica | 1950–54 | 55 | 20 |
| Barbados | 1949–54 | 672 | 11 |
| Trinidad* | 1947–55 | 2164 | 10 |
| Jamaica | 1952 | 407 | 12 |
| British Guiana | (Data not available for the different races) | | |
| England and Wales | 1955 | — | 19·7 |

* The Trinidad figures include East Indians, who constitute one-third of the population.

and the female breast ; whereas in the uterus and the digestive system as a whole the mortality is higher in Antigua.

*Cancer of the stomach*

In Table VI I have compared the relative mortality from cancer of the stomach in the two sexes in Antigua. It is much commoner per 1000 cancer deaths in the male than in the female.

The standard errors of the differences between the percentages of the West Indian and the English figures for cancer of the stomach are not statistically significant, but it will be noted that throughout the islands the mortality per 1,000 male cancers is approximately twice that per 1,000 female cancers. This should be compared with England and Wales where the corresponding figures are 16·6 and 14·3.

*Average Age of Death in Women with Cancer of the Stomach as Compared with those Dying from Cancer of the Generative Organs*

Gade (1931) raised an interesting point when he argued that not as many women as might have been expected die of cancer of the stomach because many cancer-disposed women have already succumbed to cancer of the uterus and ovary before they have had time to reach the age group in which cancer of the stomach normally occurs.

Gade was discussing Scandinavian women ; Scandinavian food and environmental factors differ considerably from those prevailing in the West Indies ;

TABLE VI.—*Cancer of the Stomach Expressed as a Proportion per* 100 *Total Cancer Deaths for Each Sex*

| Colony | Years | Total cancer deaths | | | Number of deaths from cancer of the stomach | | Gastric cancer deaths expressed as a percentage of total cancer deaths for each sex | | Population in 1952 in thousands |
|---|---|---|---|---|---|---|---|---|---|
| | | Male | Female | Total | Male | Female | Male (%) | Female (%) | |
| Leeward Islands— | | | | | | | | | |
| Antigua | 1887–1957 | 391 | 1091 | 1410 | 101 | 135 | 26 | 13 | 48 |
| St. Kitts | 1950–57 | 112 | 258 | 370 | 32 | 39 | 29 | 15 | 51 |
| Monserrat | | No data available in reports | | | | | | | 13 |
| Windward Islands— | | | | | | | | | |
| Grenada | | No data available in reports | | | | | | | 82 |
| St. Vincent | | No data available in reports | | | | | | | 72 |
| St. Lucia* | 1951–56 | 27 | 43 | 70 | 16 | 8 | 59 | 19 | 83 |
| Dominica | 1950–55 | 54 | 55 | 109 | 26 | 5 | 48 | 9 | 57 |
| Barbados | 1951–54 | 228 | 429 | 657 | 91 | 88 | 40 | 21 | 219 |
| Trinidad† | 1947–55 | 1309 | 2164 | 3473 | 409 | 298 | 31 | 14 | 663 |
| Jamaica | 1952 | 322 | 407 | 729 | 105 | 67 | 33 | 17 | 1457 |
| British Guiana | 1952–54 | — | — | 120 (in negroes only) | No data available in reports | | | | 163 (negroes only) |
| England and Wales | 1955 | — | — | — | — | — | 16·3 | 14·3 | — |

* Excluding 1953.
† The Trinidad figures include East Indians, who constitute one-third of the population.

however, I have compared the ages at death of women dying of the disease affecting their generative organs with the ages of those dying of cancer of the stomach. There can be little or no influence of one type of cancer on the other in so far as Antigua is concerned, because among the 199 women dying of cancer of the generative organs between 1920 and 1957, the average age of death was 49·3 years, (standard deviation of 13·0), and of the 72 who died in the same period from cancer of the stomach, the average age was 54·4 years, with a standard deviation of 13·2. The values of the standard deviations prevent us from assuming that there is any significant difference in the ages of death of the two conditions so far as Antigua is concerned.

### *Cancer Deaths in Relation to Deaths from all Causes*

In Antigua the proportion of deaths from cancer to those from all causes is shown for each decade from 1887 to 1957 in Table I. the proportion has steadily risen over the years. Much of the increase is due to the improved death rates, especially in infancy, in recent decades, but it is also due to better diagnosis—to the salvaging of cancers from the terms such as " old age " mentioned in an earlier paragraph. It is also due to a larger number of people living into the cancer age groups in recent times. It may be that, if cancer incidence is proved to be on the increase in the negro, this factor also plays a part.

### *Cancer Death Rate in the West Indies*

The following are the cancer death rates from neighbouring Caribbean islands. In general they corroborate the evidence collected in Antigua. In those instances where the death rate appears to be unduly low, one would suspect either that cancers are being missed or that not all deaths are medically certified, or both.

TABLE VII.—*Crude Death Rate from Cancer per* 100,000 *in the West Indian Coloured Population*

| | | |
|---|---|---|
| Leeward Islands— | | |
| St. Kitts | 1918–19 | 80 |
| | 1924–25 | 173 |
| | 1928–35 | 54 |
| | 1938–47 | 75 |
| | 1948–57 | 84 |
| Montserrat | 1947–49 | 80 |
| Windward Islands— | | |
| Grenada | 1927–33 | 45 |
| | 1949–53 | 46 |
| St. Lucia | 1948–56 | 20 |
| St. Vincent | 1945–51 | 27 |
| Barbados | 1946–54 | 78 |
| Trinidad* | 1931–40 | 41 |
| | 1941–50 | 49 |
| | 1951–55 | 64 |
| Jamaica† | 1950 | 69 |
| | 1951 | 66 |
| | 1952 | 71 |
| British Guiana (negro population only) | 1932–38 | 45 |
| | 1952–54 | 23 |

* One-third of the population are East Indians.
† About one-third of all deaths are not medically certified.

### *Varieties of Neoplasm*

The Antigua death reports do not help much in differentiating the various types of neoplasm other than carcinoma and sarcoma. An analysis of the last 320 deaths from malignant growth, all having occurred since 1948, reveals that there were six, or less than 2 per cent, due to sarcomas. This compares with a ratio of one to seventeen met with in Europe (Vint, 1935). Table VIII shows the rates that have been met with in the coloured people:

TABLE VIII.—*Cancer death Rates in Coloured People*

| Author | Country | Per cent due to carcinomas | Per cent due to sarcomas | Carcinoma : sarcoma ratio | Remarks |
|---|---|---|---|---|---|
| Hartz (1950) | Curaçao, N.W.I. | 10·2 | 1·4 | 7 : 1 | 2000 autopsies. |
| Jonchère (1948) | French West Africa | 69 | 19·5 | 3·5 : 1 | Microscope examination of 615 tumours. |
| Choisser (1929) | Haiti | 3·4 | 0·5 | 7 : 1 | 700 consecutive autopsies of which 27 were malignant tumours. |
| Smith and Elmes (1934) | Nigeria | 45 | 44 | 1 : 1 | 445 malignant tumours. |
| Vint (1935) | Kenya | 51 | 34 | 1·5 : 1 | 561 malignant tumours. |
| This paper | Antigua | 98·1 | 1·9 | 50 : 1 | 320 malignant tumours. |

The carcinoma-sarcoma ratio as met with in Antigua is very different from what has been met with elsewhere in Africans or people of Africa descent, and I am not prepared to offer a reason for it.

### *Sex Ratio of Malignant Neoplasms*

Table IX shows the sex ratio of cancers in so far as the West Indies is concerned; cancer deaths occur about twice as frequently in females as in males throughout the area.

In many of the territories adult females out-number adult males. This is nowhere so marked as in Antigua where the ratio is 1,000 to 754, but this disparity in the numbers of the sexes is not sufficient materially to affect the general conclusion about the sex ratio of cancer mortality.

TABLE IX.—*Sex Incidence of Deaths from Cancer in the British Caribbean*

| Territory | Years | Males, number and percentage | Females, number and percentage |
|---|---|---|---|
| Antigua | 1887–1957 | 319 (27·7) | 1019 (72·3) |
| St. Kitts | 1949–57 | 112 (30·3) | 258 (69·7) |
| Montserrat | (Reports do not record the sex) | | |
| Grenada | 1949–53 | 61 (32·4) | 127 (67·6) |
| St. Vincent | (Reports do not record the sex) | | |
| St. Lucia | 1948–56 (excluding 1953) | 49 (36·6) | 85 (63·4) |
| Dominica | 1950–54 | 54 (49·1) | 55 (50·9) |
| Trinidad | 1947–55 | 1309 (37·7) | 2164 (62·3) |
| Jamaica | 1952 | 322 (44·1) | 407 (55·9) |
| Barbados | 1948–54 | 373 (32·0) | 796 (68·0) |
| British Guiana | (Data not available in reports) | | |

### SUMMARY AND CONCLUSIONS

A survey of all death certificates in Antigua has been made during an investigation of the cancer death rate in the coloured inhabitants of the island. It revealed that 1410 deaths since 1887 have been due to malignant disease, which when calculated as deaths per 100,000 living showed a rate of about one third of that prevailing in England and Wales at present.

Cancer has a higher mortality among females per 1,000 at ages than among males in Antigua, especially during the later child-bearing years.

Cancer of the uterus and of the digestive system is commoner per 1,000 cancer deaths in Antigua than in England and Wales, whereas in the respiratory system and in the female breast it is not so common in the Colony.

A comparison of the data from Antigua with similar material from adjacent Colonies, although the latter is scanty and incomplete, indicates that there is little difference between the total cancer death rate, the death rates from cancer of the stomach, uterus and of the breast severally in Antigua on the one hand, and in the neighbouring territories on the other.

The sex ratio of cancer deaths is two females to one male ; this appears to be the ratio throughout the British West Indies.

The carcinoma sarcoma ratio in Antigua, 50 to 1, is very much higher than other published reports of the ratio in the negro.

The death returns do not suggest that cancer has been increasing much in this part of the West Indies during the last half century, though it is possible that there may have been an increase in late middle life.

This paper is one of several by the author assisted by a grant from the Standing Advisory Committee for Medical Research in the British Caribbean, for which the writer wishes to express his thanks.

### REFERENCES

CHOISSER, R. M.—(1929) *Nav. med. Bull., Wash.*, **27**, 551.
DELBET, P.—(1936) *Bull. Acad. Méd., Paris*, **115**, 483.
DES LIGNERIS, M.—(1936) *S. Afr. med. J.*, **10**, 478.
ELMES, B. G. T. AND BALDWIN, R. B. T.—(1947) *Ann. trop. Med. Parasit.*, **41**, 321.
GADE, F. G.—(1931) *Norsk. Mag. Laegevidensk.*, **92**, 593.
HARTZ, P. H.—(1950) *Docum. neerl. indones. Morb trop*, **2**, 161.
HASLAM, F. J. C.—(1926) *J. Hyg.*, **25**, 227.
HELLER, J. R., CUTLER, S. J. AND HAENZEL, W. M.—(1955) *J. Amer. med. Ass.*, **159**, 1628.
HIGGINSON, J. AND OETTLÉ, A. G.—(1957) *Acta Un. int. Cancr.*, **13**, 949.
HOFFMAN, F. L.—(1931) *Amer. J. Surg.*, **14**, 229.
JONCHÈRE, H.—(1948) *Bull. méd. A.O.F.*, **5**, 247.
KENNAWAY. E. L.—(1944) *Cancer Res.*, **4**, 571.
MINISTRY OF HEALTH.—(1955) The report of the Ministry of Health, Part 2. On the state of public health. London.
*Idem.*—(1955) (2) The report of the Ministry of Health, Part 2. On the state of public health. London, p. 114.
SMITH, E. C. AND ELMES, B. G. T.—(1934) *Ann. trop. Med. Parasit.*, **28**, 461.
VINT, F. W.—(1935) *Lancet*, ii, 628.
WILCOCKS, C.—(1932) *E. Afr. med. J.*, **9**, 88.
BARBADOS.—Annual Reports of the Registrar on the Vital Statistics. Bridgetown.

British Guiana.—Annual Reports of the Registrar General. Georgetown.
Dominica.—Reports of the Medical and Sanitary Departments. Roseau.
Grenada.—Reports and General Abstracts of the Registrar General. St. Georges.
Jamaica.—Annual Report of the Registrar General for the year 1952. Kingston, p. 36.
Montserrat.—Annual Reports of the Medical Department. Plymouth.
St. Kitts.—Annual Reports of the Medical Department. Basseterre.
St. Lucia.—Annual Reports of the Registrar General (Medical Department). Castries.
St. Vincent.—Annual Administration Reports of the Colony of St. Vincent. Kingstown.
Trinidad.—Central Statistical Office. Population and Vital Statistics. Annual Report for the year 1955. Port of Spain.